# Early bilingual experience is associated with change detection ability in adults

**DOI:** 10.1038/s41598-021-81545-5

**Published:** 2021-01-22

**Authors:** Dean D’Souza, Daniel Brady, Jennifer X. Haensel, Hana D’Souza

**Affiliations:** 1 Faculty of Science and Engineering, Anglia Ruskin University, Cambridge, UK; 2 School of Psychology and Clinical Language Sciences, University of Reading, Reading, UK; 3 Department of Ophthalmology, Stanford University School of Medicine, Palo Alto, CA USA; 4 Centre for Brain and Cognitive Development, Birkbeck, University of London, London, UK; 5 Department of Psychology & Newnham College, University of Cambridge, Cambridge, UK

**Keywords:** Psychology, Human behaviour

## Abstract

To adapt to their more varied and unpredictable (language) environments, infants from bilingual homes may gather more information (sample more of their environment) by shifting their visual attention more frequently. However, it is not known whether this early adaptation is age-specific or lasts into adulthood. If the latter, we would expect to observe it in adults who acquired their second language early, not late, in life. Here we show that early bilingual adults are faster at disengaging attention to shift attention, and at noticing changes between visual stimuli, than late bilingual adults. In one experiment, participants were presented with the same two visual stimuli; one changed (almost imperceptibly), the other remained the same. Initially, participants looked at both stimuli equally; eventually, they fixated more on the changing stimulus. This shift in looking occurred in the early but not late bilinguals. It suggests that cognitive processes adapt to early bilingual experiences.

## Introduction

Experiences such as musical instrument and video game training are known to modify cognitive control processes^1,2^. So, claims that practising more than one language increases cognitive demands, and thus cognitive control, come as no surprise^3^. Because words in both lexicons are activated during language use^4–6^, it is possible that bilinguals rely on domain-general cognitive control processes to monitor and inhibit the activation of words in the non-target language *when speaking*^7^. This ‘bilingual advantage’ in cognitive control has even been reported in children as young as 7 months^8^. In Kovács and Mehler’s pioneering study^8^, infants raised in bilingual homes inhibited a learned response, but infants raised in monolingual homes did not. However, 7-month-old infants do not produce words, so they do not need to inhibit words in one language in order to produce words in the other^9^. Moreover, when we tried to replicate Kovács and Mehler’s study but with a larger sample, we could not^10^. In our preregistered study^10^, infants from bilingual homes did indeed inhibit the learned response—but so too did infants from monolingual homes. Furthermore, recent meta-analyses and reviews of the adult literature call into question the strength—or veracity—of the bilingual advantage. They suggest that the bilingual advantage is rare (found in only 14.7% of all comparisons), barely discernible (*g* < 0.12), and may altogether disappear when publication bias is taken into account^11–14^.

Given these controversial findings, many researchers (e.g. Ref.^14^) argue that adaptations to language are specific to language rather than to cognition more generally. Does this mean that, unlike musicians versus non-musicians, we should not expect to find any (non-language) differences in the cognitive domain between bilinguals and monolinguals? We have argued that exposure to more varied, less predictable environments drive infants to minimise uncertainty by sampling more of their environment for supplementary visual information^9^. Because the bilingual environment may be more varied and less predictable than the monolingual environment (see Ref.^15^ for discussion), we hypothesised that infants from bilingual homes would switch attention between visual stimuli more frequently, and more rapidly disengage attention from one stimulus in order to shift it to another, than infants from monolingual homes. Our data provided evidence in favour of both these hypotheses^10^. But rather than label it as a ‘bilingual advantage’ as convention dictates, we suggested it was an adaptation to a particular set of constraints—i.e. to a more varied and unpredictable environment. It is not necessarily a lasting ‘advantage’, because constraints change over time. For example, an infant may rely on visual information (such as facial expressions, hand gestures, or lip movements) to disambiguate speech sounds, but an older child or adult could simply use sentential context or ask speakers for clarification.

If we should be looking for adaptations to particular sets of constraints, rather than searching for bilingual ‘advantages’ per se, it raises the question of whether the adaptations we found in early development (rapid attentional disengagement and more frequent attention-switching) are age-specific or persist through to adulthood. This is the focus of the current paper. We would like to know whether the early adaptations we found in infants can be found in adults. To this end, we administered the same tasks but to a different population: bilingual and monolingual adults. Our preliminary aim was to find out whether it is possible to detect a bilingual adaptation in adults: do bilingual adults, like bilingual infants, disengage attention faster and switch attention more frequently than their monolingual peers? But because we are particularly interested in whether the early adaptation persists through to adulthood (rather than reflecting a difference between bilingual and monolingual groups more generally), our primary aim was to investigate the effects of bilingual experience on attention. Specifically, we wanted to compare bilingual adults who (like the infants in^10^) had been exposed to two or more languages from an early age with bilingual adults who had learned their second language much later in life.

## Methods

We followed the same protocol as the one in the registered report^10^, but this time we compared bilingual and monolingual adults, and investigated the role of bilingual experience.

### Participants

Data were collected from 127 adults, of whom 92 were bilingual (mean age = 24.82 years, *SD* = 6.18, 69.6% women) and 35 were monolingual (mean age = 24.20 years, *SD* = 5.97, 69.7% women). The bilingual and monolingual groups did not significantly differ on either age (*t*(125) = 0.51, *p* = 0.614, *d* = 0.10) or gender (*x*^2^(1) < 0.01, *p* = 0.989).

We also obtained from each participant a socioeconomic status (SES) score. The SES score was a composite score based on self-report measures of education and household income. The education score ranged from 1 (no formal education) to 7 (doctorate or equivalent). The household income score ranged from 1 (less than £14,000 per year) to 11 (over £120,000 per year). These scores were converted into proportions (0.00–1.00). The mean education scores of the bilingual and monolingual groups were 0.65 (*SD* = 0.20) and 0.62 (*SD* = 0.14), respectively. These scores indicate an average ‘vocational’ level of education (diploma, certificate, BTEC, NVQ level 4, etc.) in both groups, *t*(125) = 0.59, *p* = 0.554, *d* = 0.12. The mean household income scores of the bilingual and monolingual groups were 0.37 (*SD* = 0.26) and 0.44 (*SD* = 0.24), respectively. These scores indicate that average household income was slightly lower (£20,001-£25,000) in the bilingual group than the monolingual group (£20,001-£30,000), *t*(125) = 1.36, *p* = 0.178, *d* = 0.27. The SES composite score (which ranged from 0.00 to 1.00) was the mean average of the education and household income scores. SES did not significantly differ between the bilingual (*M* = 0.51, *SD* = 0.18) and monolingual (*M* = 0.53, *SD* = 0.15) groups, *t*(125) = 0.68, *p* = 0.496, *d* = 0.14.

### Materials and procedure

All experimental procedures were in accordance with the Declaration of Helsinki^16^ and approved by the Faculty Research Ethics Panel of the Faculty of Science and Engineering in Anglia Ruskin University. Informed consent was obtained from all research participants.

Four eye-tracking experiments were carried out (see Ref.^10^, for details). Two of the four experiments are not relevant to the current paper, because they did not yield differences between the infant groups^10^. All experiments involved a Tobii Pro TX300 remote eye tracker to capture moment-to-moment point of gaze at a sampling rate of 120 Hz, with measurement accuracy of 0.4° (screen size: 58.42 cm; aspect ratio: 16:9; screen resolution: 1920 × 1080). The tracking equipment and stimulus presentation were controlled using customized scripts in MATLAB R2013a. Participants sat in a dimly lit featureless room, with their eyes at approximately 65 cm from the stimulus-presentation screen, and were asked to watch the display. A 5-point calibration was used. If calibration was good for at least 3 of the 5 points (e.g. precision and accuracy within 1.5° and 5°, respectively; Tobii Technology AB, 2011), then the eye-tracking experiments began. If not, then further attempts at calibration were made until calibration was good.

#### Experiment 1: the gap-overlap task

The gap-overlap task (adapted from Ref.^17^) measures the ability to disengage attention from one visual stimulus and shift it towards a different visual stimulus.

Participants were presented with three trial conditions: baseline, gap and overlap. Each trial began with a centrally presented 2.6° × 2.6° cartoon (the central fixation stimulus or central stimulus). In the baseline and gap trials, once the participant fixated on the central stimulus, the central stimulus vanished after 0.6–0.7 s. On its disappearance, an animated 2.6° × 2.6° peripheral target was immediately presented in the baseline trials and after a 0.2 s delay in the gap trials. In the overlap trials, the central stimulus did not disappear; instead it ceased flickering, but remained onscreen and overlapped with the appearance of the target. It ceased flickering so the dynamic peripheral target was more attention-grabbing. The target was presented to either the left or the right of the central fixation stimulus at an eccentricity of 14.9°. It remained onscreen until either the participant looked at it, or until 3 s had elapsed. If the participant looked at it within 1.2 s, she/he was ‘rewarded’ by one of six 2.6° × 2.6° animated cartoons (which appeared in place of the target). The time it took for the participant to shift his or her gaze to the peripheral target from the onset of the peripheral target was measured for each trial. Trials were presented in blocks of 12 until 12 ‘valid’ trials per condition were acquired or a maximum of 60 trials were presented. Trials were automatically coded ‘valid’ if the participant fixated on the target after 0.2 s and before 1.2 s of its appearance. If the participant failed to shift their gaze away from the central fixation stimulus within this time window, then the trial was automatically recorded as ‘invalid’. In addition, trials were considered invalid if the participant failed to look at the central stimulus prior to the presentation of the target or if the participant blinked or did not gaze towards the target.

To test our prediction that bilinguals are faster at disengaging visual attention than monolinguals, disengagement and gap effects were calculated by (1) subtracting saccadic RTs in the baseline condition from saccadic RTs in the overlap condition (the disengagement effect) and (2) subtracting saccadic RTs in the gap condition from saccadic RTs in the overlap condition (the gap effect). The disengagement and gap effects are two different ways of measuring the ability to disengage attention. According to Petersen and Posner^18^, selecting and attending to the relevant aspects of visual environment involve *disengaging* attention from the current focus, *shifting* to a new target, and *engaging* the new focus of attention. Therefore, the time it takes a participant to redirect their gaze from the central fixation stimulus to the peripheral target is assumed to be the time needed for the participant to disengage attention from the central fixation stimulus and shift it to, and engage, the target stimulus. Thus, in overlap trials, saccadic RT (from the onset of the target stimulus to the first look to the target stimulus) equals the time it takes to disengage, shift, and engage attention:

$${\text{RT}}_{{{\text{overlap}}}} = {\text{ RT}}_{{{\text{disengage}}}} + {\text{ RT}}_{{{\text{shift}}}} + {\text{ RT}}_{{{\text{engage}}}}$$

By contrast, if the offset of the central fixation stimulus occurs at the onset of the peripheral target, it may be assumed that the participant does not need to disengage from the central fixation stimulus before shifting attention to the target. In these ‘baseline’ trials, the time it takes the participant to shift attention to the target is arguably equal to the time it takes to shift and engage attention. Thus:

$${\text{RT}}_{{{\text{baseline}}}} = {\text{ RT}}_{{{\text{shift}}}} + {\text{ RT}}_{{{\text{engage}}}}$$

On this view, by subtracting RT_baseline_ from RT_overlap_, it is possible to derive RT_disengage_. However, it could also be argued that the processing of visual information does not stop the moment a visual stimulus disappears. A participant may require time to fully disengage their attention before shifting it elsewhere, even if the central fixation stimulus has disappeared. Hence, a temporal gap (usually 0.2 s) between the offset of the central fixation stimulus and the onset of the peripheral target is often inserted to provide the participant with time to fully disengage their attentional resources. On this view, subtracting RT_gap_ from RT_overlap_ (the gap effect^19^) provides a ‘purer’ measure of disengagement than subtracting RT_baseline_ from RT_overlap_. Because findings in the bilingualism literature are controversial, we report both measures.

#### Experiment 2: the graded change detection task

This task probed whether bilinguals shift attention more frequently and are more or less sensitive to the changing details of a visual stimulus than monolinguals.

The experiment consisted of 15 trials. All trials began with one of four attention grabbers centrally positioned on a white screen. After 1 s, the attention grabber was replaced with two blue line drawings (7.9 cm wide; 7.0°), one on either side of the screen (left, right) and 13.2 cm (500 pixels, 11.6°) from the centre. The drawings remained onscreen for 5 s, after which the trial ended. In the first trial, the two drawings were identical; they were both line drawings of a man’s head. In every subsequent trial, the drawing on one of the sides (e.g. the left) was replaced with a slightly different drawing. Over the course of the 15 trials, the drawing on one side of the screen remained the same, but the drawing on the other side of the screen gradually changed (in 14 steps) from a man’s head to a woman holding flowers (see Ref.^10^, for examples). Half the bilinguals/monolinguals saw the changes occur on the left side of the screen; half saw the changes on the right.

Two Areas-of-Interest (AOIs) were defined around the line drawings. The predefined AOIs extended 1 cm around the line drawings in case the participant focused on the outer edges of the drawings and to account for small deviations in the quality of the calibration. Two measures were obtained: (1) number of times the participant switched visual attention between the two AOIs, as a proportion of time spent looking at both AOIs, and (2) proportion of time spent looking at the AOI that the novel stimuli appeared in (e.g. if for participant 1 it was the stimulus on the left side that kept changing over the course of the experiment, then for participant 1 we obtained proportion of time spent looking at the left AOI: i.e. left AOI/(left AOI + right AOI)).

#### Bilingual experience

Bilingual experience was measured using a self-report questionnaire: the Language Experience and Proficiency Questionnaire (LEAP-Q)^20^. The LEAP-Q measures ‘age of acquisition’ for each language that the individual understands. Because we were interested in early vs. late bilinguals, we obtained a ‘bilingual experience’ score by subtracting ‘age of first language (L1) acquisition’ from ‘age of second language (L2) acquisition’ (in years). Our rationale was that zero would indicate a simultaneous bilingual (someone who acquired their first and second languages early on and in parallel), a small number would indicate an early bilingual (someone who acquired their second language shortly after their first), and a large number would indicate a late bilingual (someone who acquired their second language later in life).

## Results

### Experiment 1: disengaging attention

Neither the disengagement effect nor the gap effect differed between bilingual and monolingual adults (disengagement: *t*(92) = 0.59, *p* = 0.555, *d* = 0.14; gap: *t*(93) = 1.57, *p* = 0.119, *d* = 0.36). This suggests that the ability to disengage attention does not differ between these two groups.

**Figure 1:**
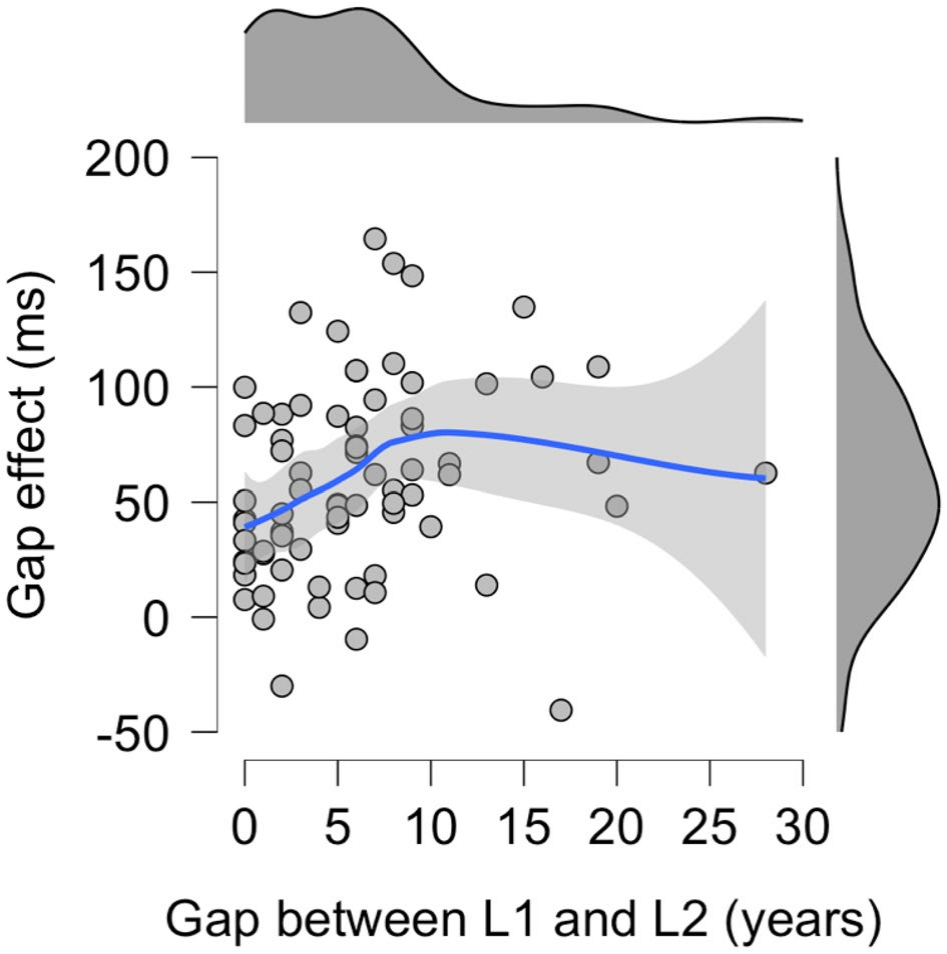
The gap effect was smaller in early bilinguals than late bilinguals. This suggests that early bilinguals disengage attention faster than late bilinguals. Bilingual experience was calculated by subtracting age of L1 acquisition from age of L2 acquisition (in years); data close to zero represent bilinguals who acquired their L2 shortly after their L1 (‘early bilinguals’), data far from zero represent bilinguals who acquired their L2 later in life (‘late bilinguals’). The grey shaded area represents 95% confidence intervals.

To quantify the relationship between the bilingual experience of an individual (the difference in years between age of L1 acquisition and age of L2 acquisition) and the corresponding disengagement/gap effect, linear, quadratic, and logarithmic regressions were performed. The nonlinear regressions were carried out because we had predicted categorical differences between early and late bilinguals. Indeed, visual inspection of the data show the expected nonlinear relationship (see Fig. 1).

Bilingual experience was not associated with the disengagement effect (linear model: *F*_1,65_ = 0.02, *p* = 0.883, *R*^2^ < 0.01; quadratic model: *F*_2,64_ = 0.53, *p* = 0.592, *R*^2^ = 0.02; logarithmic model: *F*_1,65_ = 0.42, *p* = 0.519, *R*^2^ < 0.01). However, it was associated with the gap effect. All models (linear, quadratic, and logarithmic) were statistically significant: *F*_1,66_ = 6.34, *p* = 0.014, *R*^2^ = 0.09; *F*_2,65_ = 4.22, *p* = 0.019, *R*^2^ = 0.12; *F*_1,66_ = 7.80, *p* = 0.007, *R*^2^ = 0.11; respectively. Although these are small effects (it suggests that only 9–12% of the variance in the gap effect can be explained by variation in bilingual experience), they suggest that the relationship is stronger among early bilinguals than late bilinguals.

To better understand the gap effect and bilingual experience data, we fitted a generalized additive mixed model with a 1-dimensional smooth: bilingual experience (L2-L1). Specifically, the predictor variable was entered as a thin plate regression spline^21^, which means that a smoothed function was fitted by combining several low-level functions (such as a linear function, a quadratic function, a logarithmic function, etc.) across the span of trials. Based on the Nyquist frequency, the number of low-level functions used to construct the thin plate regression spline was set to 9.

The resulting effective degrees of freedom is greater than 1 (edf = 1.38). This suggests that the effect of ‘bilingual experience’ is indeed non-linear (see Fig. 1), which needs to be taken into account. The smooth term is statistically significant, *F* = 3.31 (*Ref. df* = 1.67), *p* = 0.033 (adjusted *R*^2^ = 0.08, deviance explained = 10.1%). This supports the hypothesis that the gap effect is smaller in early bilinguals than late bilinguals.

### Experiment 2a: switching attention

In this experiment, participants were presented with two stimuli, one on either side of the screen. Over the course of 15 trials, the stimulus on one side of the screen remained the same while the stimulus on the other side changed, almost indiscernibly. Because the change in stimuli across trials is not strictly linear (i.e. some changes between trials may appear more subtle than others), we fitted a generalized additive mixed model to the data, with fixed effects of ‘trial’ (1–15) and ‘group’ (bilingual, monolingual), and a random effect of ‘participant’, and with ‘number of switches per s’ as the outcome variable. The ‘trial’ variable was entered as a thin plate regression spline^21^. Based on the Nyquist frequency, the number of low-level functions used to construct the thin plate regression spline was set to 7. Random variability in nonlinear patterns across participants was also modelled (i.e. complex nonlinear trajectories were allowed to differ between participants, so participants were not assumed to behave exactly the same way with respect to the trial variable).

**Figure 2:**
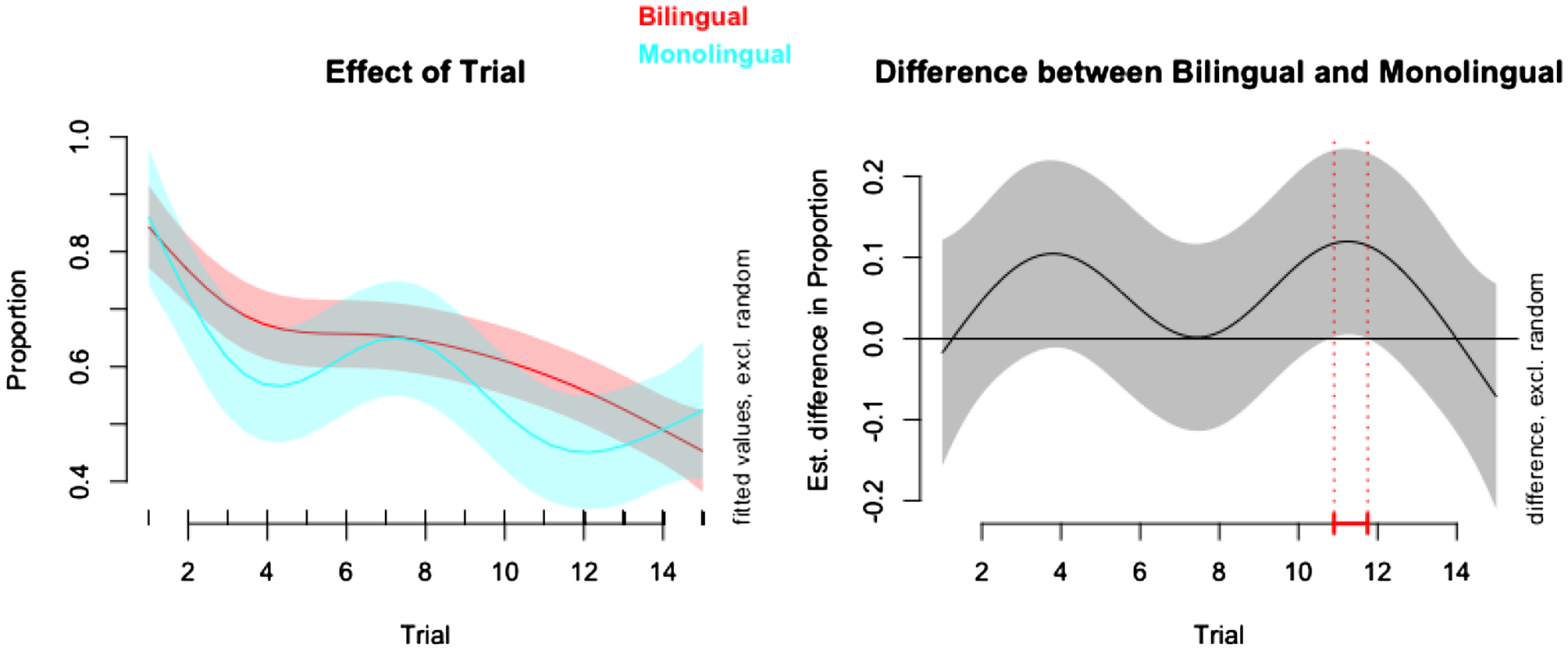
Fine grained analyses of the smooth terms in the generalized additive mixed model reveal that the estimated difference between bilinguals and monolinguals in number of switches per s (proportion of switches to time spent looking at the stimuli) is statistically significant (*p* < 0.05) around Trial 11.

In this model, the effective degrees of freedom (edf) is 4.19 for the bilingual group and 5.47 for the monolingual group (Table 1). This indicates that both smooth terms are non-linear (Fig. 2) and justifies the use of fitting a generalized additive mixed model to the data. Both bilingual and monolingual smooth terms were significant predictors of the outcome variable (both, *p* < 0.001).

**Table 1:** Approximate significance of smooth terms (outcome variable: number of switches per s).

	Edf	Ref. df	*F*	*P*
s(trial): bilingual	4.19	4.86	11.11	< 0.001***
s(trial): monolingual	5.47	5.84	6.76	< 0.001***
s(trial, participant)	316.29	852.00	1.66	< 0.001***

Parametric coefficients: intercept = 0.63 (*SE* = 0.02, *t* = 26.81, *p* < 0.001); monolingual = − 0.05 (*SE* = 0.05, *t* = − 1.06, *p* = 0.287).

GCV = 0.09, adjusted *R*^2^ = 0.48, deviance explained = 57.2%.

***p < 0.001.

To isolate the effect of ‘group’, we compared the group model with a pared down version that did not include group effects (the ‘null’ model). The difference in Akaike information criterion (AIC) was statistically significant (*χ*^2^(3) = 4.17, *p* = 0.039), which indicates that the group model (AIC = 771.89) was a better fit of the data than the null model (AIC = 776.06). (The lower the AIC, the better).

To quantify the relationship between bilingual experience and switching behaviour, we fitted a generalized additive mixed model to the data, with a fixed effect of ‘trial’ (1–15) and random effects of ‘participant’ and ‘bilingual experience’, and with ‘number of switches per s’ as the outcome variable. As before, the ‘trial’ variable was entered as a thin plate regression spline^21^, with the number of low-level functions set to 7. Random variability in nonlinear patterns across participants was also modelled.

In this model, the effective degrees of freedom of the bilingualism smooth term is 1.00, indicating that any relationship is linear. Although the bilingual experience model (AIC = 363.54) is a better fit of the data than the null model (AIC = 366.84), the difference is not statistically significant: *χ*^2^(3) = 3.30, *p* = 0.086.

### Experiment 2b: noticing change

To ascertain whether the bilingual adults noticed the changes before the monolingual adults, we ran the same analyses as in the previous section, but this time the outcome variable was ‘proportion of time looking at the changing stimulus to time looking at both stimuli’. The generalized additive mixed model included fixed effects of ‘trial’ (1–15) and ‘group’ (bilingual, monolingual), and random effects of ‘participant’. The ‘trial’ variable was entered as a thin plate regression spline^21^ and the number of low-level functions used to construct the thin plate regression spline was set to 7. Random variability in nonlinear patterns across participants was also modelled.

**Figure 3:**
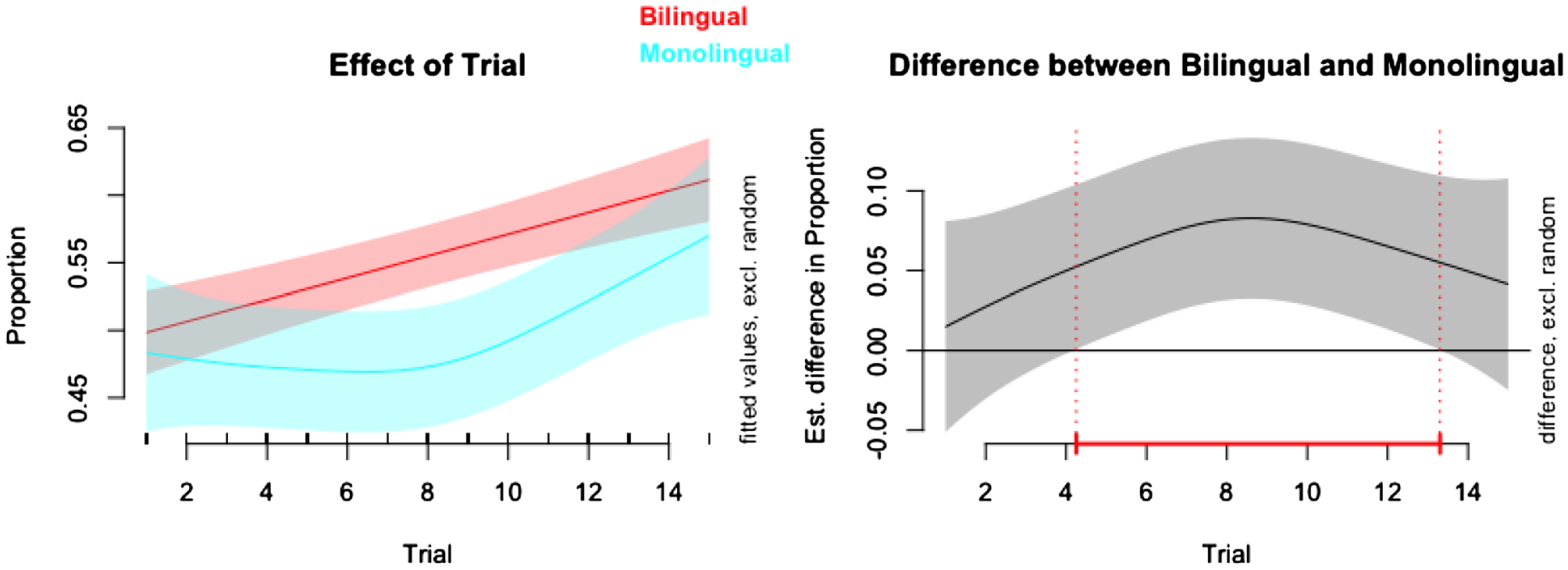
Analysis of smooth terms in a generalized additive mixed model reveals that the difference between bilinguals and monolinguals in proportion of looking at the changing stimulus is statistically significant (*p* < 0.05) between Trials 5 and 13. This suggests that the bilingual group noticed the changing stimulus earlier than the monolingual group. However, the model is not an improvement on a pared down version of the model which contains no group terms. We must therefore interpret these findings with caution.

In this model, the effective degrees of freedom (edf) is 1.00 for the bilingual group and 2.11 for the monolingual group. This indicates that the bilingual smooth term is linear, while the monolingual smooth term is non-linear (Fig. 3). This justifies fitting a generalized additive model to the data. Both bilingual (*p* < 0.001) and monolingual (*p* = 0.010) smooth terms significantly predict proportion of looking (Table 2).

**Table 2:** Approximate significance of smooth terms (outcome variable: proportion of time looking at the changing stimulus).

	Edf	Ref. df	*F*	*P*
s(trial): bilingual	1.00	1.00	28.44	< 0.001***
s(trial): monolingual	2.11	2.58	4.08	0.010**
s(trial, participant)	167.72	851.00	0.66	< 0.001***

Parametric coefficients: intercept = 0.55 (*SE* = 0.01, *t* = 46.93, *p* < 0.001); monolingual = − 0.06 (*SE* = 0.02, *t* = 2.52, *p* = 0.012).

GCV = 0.04, adjusted *R*^2^ = 0.27, deviance explained = 34.1%.

**p < 0.01, ***p < 0.001.

To isolate the effect of ‘group’, we compared the group model with a model that did not include group effects (the ‘null’ model). As the Akaike information criterion (AIC) is lower in the null model (AIC = − 697.73) than the group model (AIC = − 696.65), the group model is not a better fit of the data than the null model. Why would the group model be no different from the null model when the group model’s smooth terms are all statistically significant? To balance good fit with parsimony, AIC penalises model complexity—and whereas the null model’s edf is 5 (low complexity), the group model’s edf is 8 (high complexity). In any case, we cannot conclude that there is a meaningful difference between bilinguals and monolinguals.

To quantify the relationship between bilingual experience and proportion of looking to the changing stimulus, we fitted a generalized additive mixed model with a fixed effect of ‘trial’ and random effects of ‘participant’ and ‘bilingualism (L2-L1) by trial’, and with ‘proportion of looking to the changing stimulus’ as the outcome variable. The trial variable was entered as a thin plate regression spline^21^ and the number of low-level functions used to construct the thin plate regression spline was set to 7. Random variability in nonlinear patterns across participants was also modelled.

**Figure 4:**
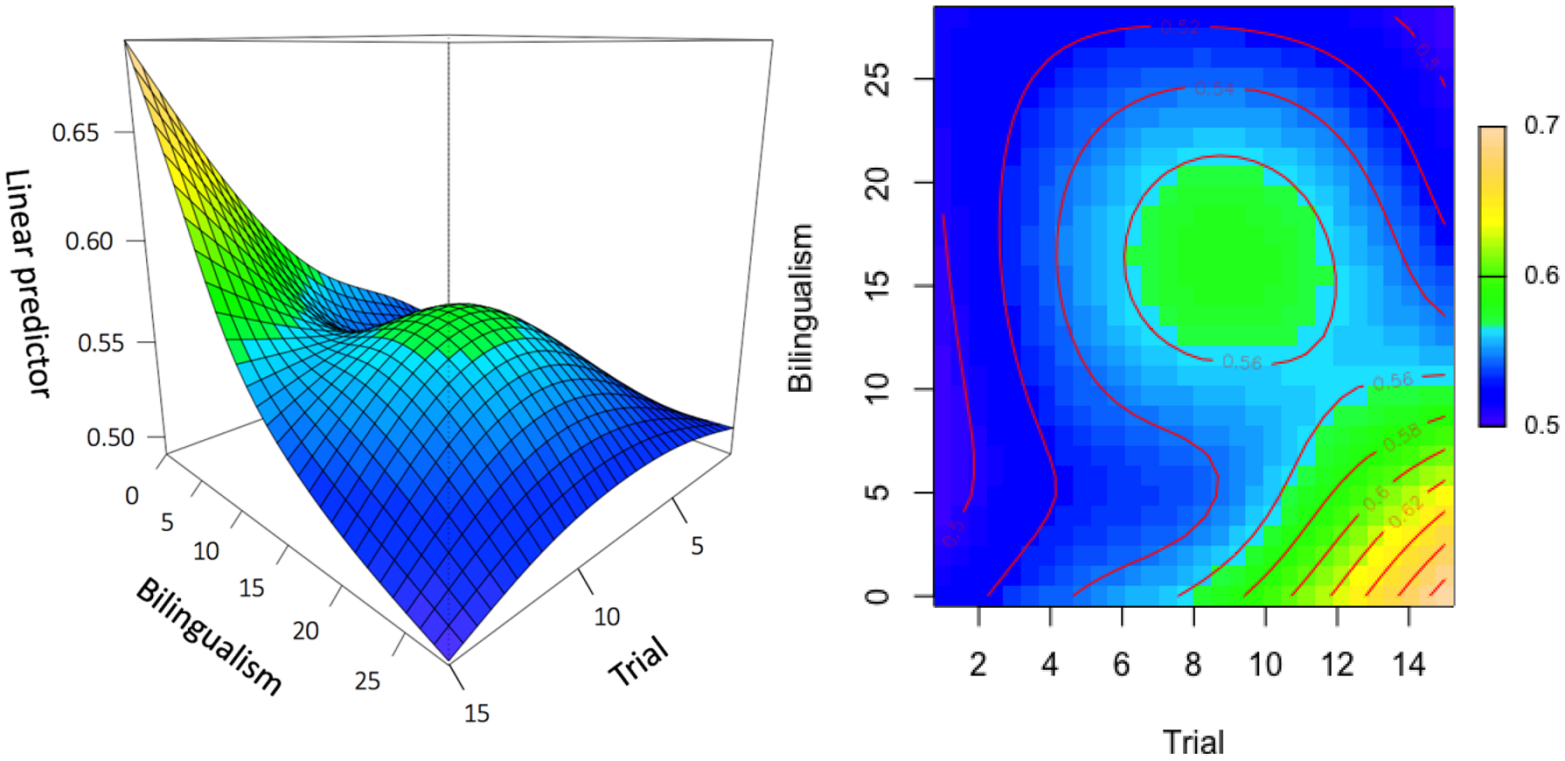
Proportion of looking to the changing stimulus (the outcome variable) rises rapidly during the final trials of the experiment for individuals who had learned their second language shortly after learning their first language—but not for individuals who had learned their second language later in life. ‘Bilingualism’ was calculated by subtracting age of first language acquisition from age of second language acquisition (in years); scores close to zero represent bilinguals who acquired their second language shortly after their first language (‘early bilinguals’), scores far from zero represent bilinguals who acquired their second language later in life (‘late bilinguals’).

In this model, the effective degrees of freedom is greater than 1 (Table 3), indicating that the effect of ‘bilingualism by trial’ is indeed non-linear (Fig. 4).

**Table 3:** Approximate significance of smooth terms (outcome variable: proportion of time looking at the changing stimulus).

	Edf	Ref. df	*F*	*P*
s(trial, participant)	102.99	621.00	0.52	< 0.001***
s(trial)	4.26	5.01	8.67	< 0.001***
ti(trial, bilingualism)	2.13	2.58	3.98	0.009**

Parametric coefficient (intercept) = 0.55 (*SE* = 0.01, *t* = 54.52, *p* < 0.001).

GCV = 0.03, adjusted *R*^2^ = 0.24, deviance explained = 30.1%.

**p < 0.01, ***p < 0.001.

To isolate the effect of bilingual experience, we compared the bilingualism model with a version that did not include an effect of bilingual experience (the ‘null’ model). The Akaike information criterion (AIC) is lower in the bilingualism model (− 712.55) than the null model (− 705.46), indicating that the bilingualism model is a better fit of the data, *χ*^2^(3) = 7.09, *p* = 0.003.

Altogether, these data suggest that early bilinguals were better at detecting the change than late bilinguals.

## Discussion

Previously, we suggested that infants from bilingual homes adapt to their more complex language environment by sampling more of their visual environment and placing more weight on novel information^9^. This is consistent with the results of our previous study^10^ which found that infants from bilingual homes disengage attention faster from one stimulus and switch attention more frequently between stimuli than infants from monolingual homes. The aim of the present study was to ascertain whether the early adaptations we found in infants^10^ could also be found in adults. We found that early bilingual adults may indeed disengage attention faster than late bilingual adults. This raises the possibility that an adaptation found in infants exposed to bilingual homes extends to adulthood and is not found among late bilinguals.

However, the early bilinguals did not switch attention more frequently (per second) than the late bilinguals. This suggests that the tendency to switch attention more frequently is specific to certain periods of development. Why would this be the case? One explanation is that cognitive control increases during the early stages of bilingualism^22^ but then plateaus at peak efficiency^23^ as processing becomes automatised^24^. But this would not explain why the early bilinguals were faster at disengaging attention than the late bilinguals. An alternative explanation is that development of the visuomotor system asymptotes towards an optimal solution. In other words, the visuomotor system becomes so efficient at processing the features of simple stimuli and switching attention between two nearby stimuli that individual differences become negligible by early adulthood. On the other hand, disengaging attention and detecting gradual change may require higher level processes that are far from optimal, even in adults. A different line of argument is that tendencies (i.e. the *likelihood* of behaving in a particular way, such as switching attention) are more modifiable than abilities (the *potential* to do something, such as the speed at which one can disengage attention when orienting to a new stimulus). In other words, the speed at which one can disengage attention may be more stable over developmental time than the tendency to explore. A fourth possibility is that different mechanisms operate at different points in development^9^. For example, whereas bilingual children show more of a general executive control advantage, bilingual adults show more of an inhibitory control advantage^12^. To elucidate the pertinent mechanisms, large-scale studies that relate different aspects of bilingualism (e.g. age of acquisition, similarity of languages spoken, language proficiency, patterns of language use^25^) to nonverbal cognitive tasks across developmental time (from infancy to old age) need to be carried out.

If it is true that infants from bilingual homes adapt by erring on the side of visual exploration, then one might predict that they (and early bilingual adults) would be better at detecting changes in the visual environment. Unfortunately, neither the bilingual infants nor the monolingual infants seemed to detect the changes in our challenging graded change detection task^10^, but interestingly—and quite unexpectedly—we found that the early bilingual adults were indeed better at detecting the changes than the late bilingual adults. This could be because the early bilinguals had adapted to their more complex language environments by erring on the side of visual exploration^10^.

However, if there is a link between early visual exploration and detecting change, why did the early bilingual adults not switch attention more frequently per second than the late bilingual adults? It is possible that switching attention between two simple stimuli more than a few times per trial is not a particularly useful skill for adults. To detect the changing stimuli in the graded change detection task, participants had to inspect two visual stimuli, after which, and after a 1 s gap, they had to inspect another two visual stimuli while the representations of the initial stimuli fade. In other words, to succeed on this task, participants must *simultaneously* represent two complete (detailed) visual representations—and then another two. This is challenging^26^. There may not have been any evolutionary pressure to detect the kinds of changes that occurred in this experiment. Indeed, some studies suggest that detailed representations last only 0.5 s or until a new stimulus replaces them^26,27^. Nevertheless, we speculate that this is something that early bilinguals have had practice with. We have argued that children raised in more complex language environments minimise uncertainty by actively seeking out multiple sources of information: a mouth movement, a facial expression, a subtle gesture^10^. They would need to simultaneously construct—and inspect—several visual stimuli, in order to discern their meaning and match the visual information to the auditory information. Perhaps this is a skill that monolinguals and late bilinguals never need to develop to the same extent as early bilinguals. It is something we would like to examine in a future study.

In summary, we found that unlike infants from bilingual homes, early bilingual adults do not switch attention more frequently. However, early bilingual adults do appear to be faster at disengaging attention and detecting the difference between two visual stimuli than late bilingual adults. This may not be a trivial skill, because even changes to *attended* stimuli frequently go unnoticed^28,29^—and we found a difference with only two stimuli present. We speculate that early bilinguals had more experience of disengaging attention and simultaneously constructing two or more complete (detailed) visual representations, and that these are not abilities that late bilinguals develop to the same extent. If this is true, then it suggests that traces of early adaptations to constraints in the language environment are detectable as late as adulthood.

## Data Availability

The datasets generated during the current study are available in the Open Science Framework repository, osf.io/qpfyk.
